# The Mental Health of Black Youth Affected by Community Violence: Family and School Context as Pathways to Resilience

**DOI:** 10.3390/children9020259

**Published:** 2022-02-15

**Authors:** Donte T. Boyd, Kristian V. Jones, Camille R. Quinn, Adrian Gale, Ed-Dee G. Williams, Husain Lateef

**Affiliations:** 1 College of Social Work, The Ohio State University, Columbus, OH 43210, USA; quinn.395@osu.edu; 2 School of Social Work, University of Washington, Seattle, WA 98105, USA; kjones21@uw.edu; 3 School of Social Work, Rutgers University, New Brunswick, NJ 08901, USA; adrian.gale@rutgers.edu; 4 School of Social Work, University of Michigan, Ann Arbor, MI 48109, USA; eddeew@umich.edu; 5 Brown School, Washington University in St. Louis, St. Louis, MO 63130, USA; hlateef@wustl.edu

**Keywords:** mental health, community violence, families, black youth, resilience

## Abstract

Black youth who experience community violence occupy multiple environments with varying levels of influence on how they display resiliency to prevent adverse mental health outcomes. Considering the recent rise of mental health concerns (i.e., increase in suicidal outcomes) among Black youth, along with the abundance of research illustrating the detrimental impact of community violence, more research is needed to examine how different environmental factors (e.g., family and school) shape how youth protect their mental health while displaying resiliency navigating community violence. The purpose of this study was to examine how family and school contexts predict Black youths’ ability to display resiliency to navigate community violence and prevent adverse mental health outcomes. This study utilized a path analysis to examine the associations between parent relationships, parent bonding, school climate, resilience to adverse community experiences, community violence, and mental health among 548 Black adolescents in Chicago. Findings highlight that parent relationships, parent bonding, and school climate influence the association between resilience to community violence and mental health outcomes among Black youth. Implications for mental health practice and policy among Black youth are discussed.

## 1. Introduction

Today, Black adolescents in the United States grapple with unique contextual and structural hurdles specific to their generation, including the rise in racial unrest due to police brutality against Black people, terrorism, crime, racism, poverty, and a global pandemic [1,2,3,4]. Generally, adolescent development is a stage that is characterized by unique stressors to their mental health [5]. However, mental health struggles of Black youth in the United States have been a cause for even greater concern. Among adolescents in the United States, suicide is the second leading cause of death [6] and among Black youth, recent research indicates a significant spike in the rate of suicide for Black children and adolescents [6].

Black youth often experience community and neighborhood factors (e.g., neighborhood poverty) that disproportionately put them at risk of being exposed to contextual factors that can have detrimental effects on their psychological wellbeing [7,8,9]. Specifically, research demonstrates that Black youth are more likely to live in low income and under-resourced communities that puts them at risk of being exposed to community violence in their neighborhoods [10,11,12,13,14]. Moreover, researchers have found community violence to be associated with detrimental mental health outcomes for youth [15,16,17].

Unfortunately, despite the current evidence that highlights the detrimental impacts of community violence for Black youth, research that examines Black youth’s resilience in navigating community violence, specifically as it relates to their mental health, is limited [18]. Focusing on the environmental context of Black youths’ resilience dealing with community violence is of the upmost importance because research highlights that not only does community violence negatively impact their personal mental health, but the repeated exposure to community violence has significant impacts on youths’ familial relationships as well as their school experiences [19].

Research highlights the importance of considering the family context of Black youth when assessing their ability to successfully navigate community violence [18]. Specifically, research demonstrates that parent support can mitigate future acts of violence for male youth who have witnessed community violence [20]. Further, family support can be a significant protective factor for youth who have been victims of community violence [21]. However, family dynamics that involve high levels of stress and instability can lead to youth who do not rely on family support when dealing with community violence [21].

In addition to general family dynamics and parent support, literature has highlighted parent bonding as an important factor to consider when investigating how youth cope with community violence. A review conducted by Ozer and colleagues [22] demonstrated that close and warm relationships with parents was a protective factor for both internalizing and externalizing symptoms among youth exposed to community violence. However, there is research which indicates the effectiveness of parent bonding is impacted by levels of exposure to community violence [23]. Specifically, greater community violence exposure has a negative effect on the protective mechanism of parent bonding. Importantly, research illustrates that youth who are exposed to higher levels of community violence have more negative perceptions of their parent [24].

With respect to school experiences and academic outcomes, research has also demonstrated that youth who are exposed to community violence are at higher risk for several lower levels of academic achievement [25]. Additionally, exposure to community violence can lead youth to display disciplinary issues in school that may likely be associated with polyvictimization, i.e., trauma, and thus lead them to involvement with law enforcement and court-related delinquency [26,27]. The study carried out by Borofsky and colleagues [28] demonstrated that community violence negatively impacted school engagement over time. Overall, previous literature emphasizes the importance of considering both the school and family context when considering community violence among Black youth.

## 2. The Present Study

As highlighted in the introduction, the context of community violence and its impact on developmental outcomes in the lives of Black youth is not a sequestered phenomenon. Their experiences are instead embedded within networks of relationships—relationships that occur within multiple environments with varying positions of influence. Given that, we used ecodevelopment theory to frame this study. Specifically, ecodevelopment offers a useful way to conceptualize how Black youth interact within different relationships based on the role of factors associated with their growth and development or lack thereof. Further, it elucidates risk but especially protective factors that function during adolescence [29]. Ecodevelopment theory is based on the creation of social ecology, which notes that youth develop based on conditional effects from four interconnected systems: (1) the microsystem (i.e., parent conversations on sexual health and drug use), (2) the mesosystem (i.e., how peers are monitored by their parents), (3) the exosystem (i.e., parent support systems), and (4) the macrosystem (i.e., culture and cultural shifts) in which they are positioned [30,31]. In addition, ecodevelopment theory suggests that youth interactions are influenced by these outer systems and the impact of their actions and views and form the matching risk and/or protective factors, including their relationships. This theory is important to center Black adolescent health outcomes due to their social status across these systems based on parent–child communication among other factors as a family system [32].

Subsequently, proposed relationships between parents and youth, school climate resilience, violence exposure, and mental health must be considered in context with multiple dimensions of influence. The present study examines how the family and school contexts influence how Black youth display resilience to protect their mental health when grappling with community violence. The present study fills a gap in the literature by examining how environmental contexts (e.g., family and school) influence how Black youth display resiliency to navigate community violence and prevent detrimental mental health outcomes. This study hypothesizes: (1) strong parent relationships will be associated with resilience to adverse experiences to community violence; (2) higher levels of parent bonding will be associated with resilience to adverse experiences to community violence (3) parent relationships will be associated with school climate; (4) and school climate will be associated with mental health.

## 3. Method

The data for this study come from the parent study, the Resilience Project collected in 2013–2014, a study examining the risk and protective mechanisms related to sexual behaviors of Black adolescents living in four urban neighborhoods of concentrated poverty in Chicago: Englewood, Woodlawn, Kenwood, and South Shore. Youth were recruited from three high schools, one youth church group, two community youth programs, and four public venues (e.g., parks and fast-food venues). The response rate for this study was 87% and the total participants for the study were 548 Black adolescents, ages 12 to 17. These participants were recruited from low-income communities consisting predominantly of Black residents where the average annual median income ranged from USD 24,049 to USD 35,946, which is below the Chicago city average of USD 43,628. The percentage of single-mother households in these areas ranged from 28.9% to 32.3%, with the city average being 13.9%.

To recruit adolescents, flyers with information regarding the study were posted at schools, community programs, and churches, where the school principals, leaders of church groups, and youth programs had permitted the researchers to recruit participants for the study. Participants were required to have both parent consent and youth assent to participate in the study. Youth who returned consent forms signed by a parent or guardian were enrolled in the study. Youth recruited in public venues were only asked to participate if a parent was present to provide consent. Trained research assistants introduced the study to all potential participants recruited from the locations with a detailed letter describing the study along with parent consent forms.

Participants recruited from schools, community programs, and churches were given a questionnaire at those respective locations. Youth who were recruited in public venues were given questionnaires in quiet spaces at or near those venues. In such instances, questionnaires were only administered to youth if a parent or a guardian was present to provide consent and the questionnaire could be immediately administered. The questionnaire took approximately 45 min to complete, after which the youth participant was given a USD 10 cash compensation. The University Institutional Review Board of the last author who collected the data approved the study.

### 3.1. Measures

The outcome variable for this study was *Mental Health* that assessed behaviors using the Brief Symptom Inventory [33,34] and contains 18 items about mental health symptoms during the past seven days (e.g., nervousness or shakiness inside, spells of terror or panic, thoughts of ending your life). Response options were based on a five-point scale (not at all, a little bit, moderately, quite a bit, or extremely). A composite mental health score was calculated by summing the responses for the 18 items. Cronbach’s alpha was α = 0.92 (range 0 to 61).

School Climate assessed school engagement. School Climate was assessed by 5 items from the modified School Bonding Scale [35]. For example, items included, “how much do you like school?” and “how much do you try in school?” Cronbach’s alpha was acceptable (α = 0.92) (range = 0–4).

Parent Bonding was measured using a 4-item scale, and the respondents were asked questions such as “how close do you feel to your father?” and “how close do you feel to your mother.” The response categories ranged from 1 = not at all to 5 = very much, and higher scores indicated an increase in parent–child relationships. The Cronbach’s alpha for this scale was 0.75 (range = 1–5) [36].

Parent Relationships was measured using a 7-item scale, and the items have been used in prior research [37,38,39]. Study respondents were asked questions such as “how disappointed would your parents be if you did not graduate from high school” and “how well do your parents know how you spend money?” The response categories ranged from 1 = not at all to 5 = very much, and higher scores indicated an increase in parent–child relationships. The Cronbach’s alpha for this scale was 0.86 (range = 1–5).

Resilience to Adverse Community Experiences to Violence (RACV) was assessed by utilizing an 10-item scale, from the Exposure to Violence Probe [40]. Participants responded to items such as “I try to attend school regularly, so that I can graduate and get out of my community”, “I try to work hard in an activity that may help me to get out of my community”, and “I try to work hard in school, so that I can get out of my community”. The response categories ranged from 1 = never to 4 = very often, and higher scores indicated an increase in resilience to adverse community experiences to violence. The Cronbach’s alpha for this scale was 0.75 (range = 1–4).

Community Violence was assessed by a single item, and asked respondents “I just accept that there is crime and violence my community” The response categories ranged from 1 = never to 4 = very often, and higher scores indicated an increase in parent–child relationships

### 3.2. Statistical Analysis

**Figure 1:**
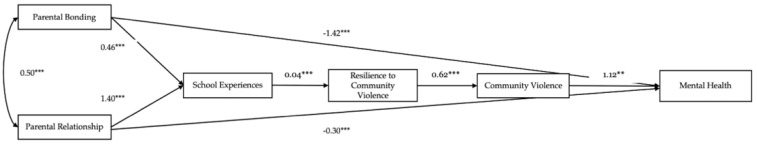
Simplified conceptual path model of mental health pathways, direct effects (single-headed arrows). *p* < 0.01 **, *p* < 0.001 ***.

**Figure 2:**
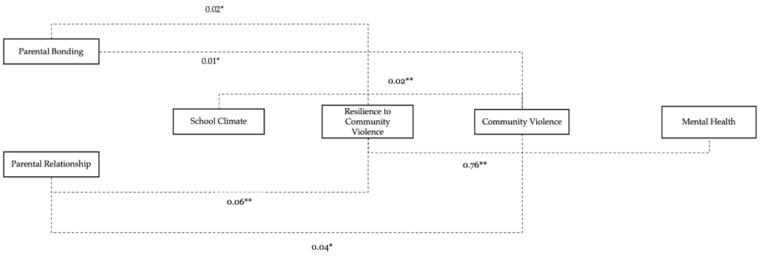
Conceptual path model of mental health pathways, indirect effects (broken arrows). *p* < 0.05 *, *p* < 0.01 **.

All analyses were conducted on observations that included non-missing data for the outcome, mental health. Statistical tests of association were conducted between measures described in the “Section 3”, including the outcome variable. Table 1 presents the descriptive statistics for this study. A bivariate correlation was conducted between the study variables (Table 2). Next, a path analysis (Table 3) examined the associations between RACV, parent bonding, parent relationships, community violence, school climate, and mental health (Figure 1, direct paths) and Figure 2 represents the path analysis with only the indirect paths. For the model generated for this study, the model fit was considered good if the χ^2^ value was non-significant, comparative fit index (CFI) > 0.95, Tucker–Lewis’s index > 0.95 (TLI), the root mean square error of approximation was ≤0.06 (adequate if ≤0.08) (RMSEA). The Akaike information criterion (AIC) and Bayesian information criterion (BIC) were used to compare the fit between the models. These fit indices were assessed as path models were generated. The Bollen–Stine bootstrap procedures with 6000 bootstrap resamples were also used to assess the consistency of the proposed model with the sample data. All analyses were carried out using STATA 17, and all statistical tests of significance accounted for the effect of weighting.

## 4. Results

### 4.1. Descriptive Statistics

Among the 636 participants, 44.6% were male and 54.4% were female, and the mean age was 15.46 years (SD = 1.12, range 12–17). Slightly more than three-fourths (74.7%) of the overall sample qualified for free or reduced school lunch, which indicates that most participants resided in low-income households. Approximately 56% of the participants lived in a single parent household. Approximately 70% reported that the climate in their school was positive. Among Black youth, 62% reported having positive relationships with their parents and 70% indicated they were working hard in school to leave their community. Lastly, approximately 20% of the sample reported mental health symptoms.

### 4.2. Bivariate Correlations

Table 2 provides bivariate correlations between the primary study variables and the outcome variable of mental health. The results of the Pearson correlation indicated that there was a significant and negative association between school climate (*r* = −0.14, *p* < 0.05) and mental health, i.e., depression symptoms. There were negative relationships between parent relationships and mental health (*r* = −0.11, *p* < 0.01) and positive relationships with school climate (*r* = 0.2, *p* < 0.001). RACV was positively correlated with school climate (*r* = 0.23, *p* < 0.001) and positively correlated with parent relationships (*r* = 0.13, *p* < 0.001).

### 4.3. Path Analysis

We conducted path analysis and the model demonstrated a good overall model fit for the sample data (χ^2^ = 5.53(6), *p* = 0.76; CFI = 0.95; TLI = 0.95; RMSEA = 0.01; AIC = 99; BIC = 100). Table 3 depicts the unstandardized and standardized results for Parent Relationships, i.e., mother bonding (N = 548). Figure 1 presents the path model (direct effects) with standardized coefficients on significant paths. Results showed that parent bonding (β = 0.46; *p* < 0.001) and parent relationships (β = 1.39; *p* < 0.001) directly predicted school experiences. Our results also indicated that school experiences directly predicted RACV (β = 0.46; *p* < 0.01). RACV directly influenced community violence (β = 0.62; *p* < 0.001). Community violence (β = 1.22; *p* < 0.001), parent bonding (β = −1.42; *p* < 0.001), and parent relationships (β = −0.29; *p* < 0.001) all directly associated with mental health.

Figure 2 shows the indirect effects of the path analysis. School climate (β = 0.03; *p* < 0.01), parent bonding (β = 0.01; *p* < 0.05), and parent relationships (β = 0.04; *p* < 0.001) indirectly influences community violence. RACV is indirectly associated with mental health (β = 0.76; *p* < 0.01). Lastly, parent relationships (β = 0.06; *p* < 0.01) indirectly influenced RACV.

## 5. Discussion

The primary aim of this study was to examine Black youths’ resilience in the face of community violence and its connection to parent relationships, school climate, and mental health. Findings from our study indicate that the influence of parent relationships and school climate are prominent regarding the mental health of Black adolescents.

Parent relationships and parent bonding are positively associated with school climate. Findings highlight a significant and negative association between Black adolescents’ parent relationships and school climate. Considering most of the participants qualified for free or reduced lunch, this finding could be linked to Black adolescents in this study sample experiencing strain associated with their parents’ lack of financial means, which could influence their experiences in school. A previous study conducted by Hopson and Lee [41] found family poverty was negatively associated with grades and behavior in school. However, the same study found students from impoverished families who perceived a positive school climate reported positive grades. This finding further emphasizes the importance of schools to consider the youth’s family context. Given this study result, the second hypothesis was partially supported by our analysis.

School Climate is positively associated with RACV. In our study, school climate was positively correlated with RACV. Consequently, school experiences as well as academic outcomes reflect the climate of the school setting overall. Youth who are exposed to community violence are at higher risk for poorer academic outcomes [25]. This is further aligned with the strong link between community violence and youth law-breaking behavior in school associated with trauma and other mental health issues, which can lead to law enforcement involvement and subsequent court involvement [26,27]. Overall, previous literature emphasizes the importance of considering both the school and family context when considering community violence among Black youth. This finding notes the need for school personnel to be educated about trauma and community violence as well as its impact on Black youth in the school and community context.

RACV is positively associated with community violence. We also found that RACV directly influenced community violence in our path analysis. Similar to our other study results regarding RACV and community violence, this study finding provides greater empirical understanding about an understudied area for Black youth [19]. Given this was a direct relationship, resilience reflects a construct that needs to be further explored as a protective mechanism, especially for youth who have histories of violence exposure.

Community violence is positively associated with mental health—parent relationships and parent bonding are negatively associated with mental health. Our study findings indicate relationships among community violence, parent bonding, and parent relationships all directly associated with mental health. This is useful as the finding contributes to the scant literature addressing the environmental context of Black youth and their resilience. Further, this finding is noteworthy because it highlights the direct association with their personal mental health as well as the impact of persistent community violence exposure on youths’ familial relationships [19].

RACV indirectly influences mental health. We noted that RACV is indirectly associated with the mental health of Black youth. Although adolescent development is known to be a point in life that is characterized by unique stressors regarding their mental health, Black youth experience challenges and difficulties that require greater concern. Most recently, severe mental health issues, such as suicide, have spiked among Black youth and children, suggesting an even more dire situation that needs to be addressed [6].

Parent relationships indirectly influence RACV. Literature suggests that the repeated exposure to community violence has significant impacts on youth familial relationships as well as their school experiences [19]. Our results suggest that higher levels of parent relationships were indirectly associated with resilience to adverse experiences to community violence. This was a surprising result and did not support the first hypothesis that resilience would directly predict parent relationship.

School Climate is negatively associated with mental health. Findings illustrate significant associations between Black adolescent’s experiences in school and their mental health. This finding is consistent with previous studies highlighting school climate is associated with the mental health of students [42]. Given this study result, the third hypothesis was supported by our analysis.

Parent relationships indirectly positively associated with mental health. Our findings suggest there were negative relationships between parent relationships and Black adolescents’ mental health. The evidence surrounding parent relationships and mental health among Black youth has been mixed in this area. Some scholars noted no statistical differences in depression based on the CES-D scale among Black youth living in public housing given their relationships with their parents [43]. However, other scholars report parent support could have an adverse impact on Black youths’ mental health. Specifically, if Black adolescents’ parents experience psychological distress, (i.e., substance misuse, incarceration, or posttraumatic stress disorder (PTSD), their mental health is more likely to worsen. Therefore, it is important for providers to enrich communication with parents and encourage them to observe when their children express feelings of sadness as well as irritability that may often mask depressive symptoms [43].

Resilience is indirectly associated with school climate. The indirect effect of Black adolescents’ resilience was positively associated with school climate, which is opposite of the finding related to parent relationships and school climate. Here, adolescents were able to thwart the adversity caused by community violence that resulted in a favorable increase in their school experiences. Past research suggests that living in communities where they experienced violence, over policing along with others forms of racial discrimination were assuaged by Black adolescents’ resilience. Moreover, the findings from this study provide further evidence that the school context can be a potential protective factor for Black youth [44].

## 6. Limitations

This study and its findings should be interpreted with the consideration of several limitations. First, the data were collected at one point in time, as cross-sectional data, so we cannot draw any temporal or causal inferences for conclusions. Second, the study focus was specific and concentrated on Black adolescents in four communities in Chicago, IL that are in concentrated poverty with high rates of community violence. Though these characterizations may align with more indicated populations—those who may be homeless or detained/incarcerated, the study findings are not necessarily generalizable to other groups of Black youth outside of this geographical location, including other urban settings. Third, the data for this study were collected several years ago, so there may have been some areas of progress made in terms of mental health services for this population, including parent relationships and dynamics, which is a limitation in this study. However, the data allowed us to investigate some of these parent–youth relationships and how they influence Black youth mental health.

Future research should seek to examine the simultaneous effects of the independent and sequential effects of community violence to statistically investigate the interaction effects between these constructs that may play an important role in how they are associated with Black adolescents’ mental health and wellbeing. Further, more empirical investigations need to be conducted with Black youth to develop a better understanding about resilience and its role among Black youth. There should also be a concerted effort to oversample understudied populations, such as adolescents who self-identify as a sexual minority, and the role of parent communication and support given the presence of adversity and isolation they experience [45]. Lastly, future research should seek to investigate the relationships among parent–child relationships, community violence, and mental health in a national representative sample of Black families.

Despite the study’s limitations, the strengths of our study lie in the use of a unique sample of Black youth in Chicago communities who have been adversely affected by social disadvantage, racism, and the lack of social capital as a unique lens to assess the mental health equity of Black adolescents. Research on positive youth development underscores that youthhood is more than risk factors and includes intertwined experiences with resiliency, strengths, and protective factors. Thus, this study adds to the knowledge base to inform prevention and intervention efforts that could serve to enhance parents and schools’ knowledge base about enhancing the resilience among Black adolescents to promote their mental health wellness.

## 7. Practice and Policy Implications

Our findings have significant implications for practice and policy associated with the mental health of Black youth. There are important practice implications related to prevention, treatment, and intervention. The direct and indirect relationships between resilience and parent–child relationships suggest that parents may be affected by community violence in different ways from their children. Recent violence prevention work based on the World Health Organization/CDC violence prevention framework has focused on cultivating best practices to develop safe, stable, and nurturing relationships between youth and their parents/caregivers [26]. This is an innovative approach that may be helpful for practitioners looking for targeted assessments and specialized services for Black youth in urban communities impacted by violence that include the parents and caregivers. Moreover, practice efforts that include parents and/or caregivers should be prioritized to incorporate pathways to healing that may curtail future parent stress and distress and thus promote resilience of Black adolescents.

Policies that “do no harm” and promote self-sufficiency are warranted, starting with violence prevention efforts that cut to the heart of the matter—poverty. Many families struggle to make ends meet and this struggle has been further exacerbated by the COVID-19 pandemic [2]. There is a need for individual and agency interaction through coalitions to organize and manage interagency, multi-disciplinary, and community-wide collaboration, and update systemic policies toward community violence prevention and intervention. Consequently, youth need services that will reduce their difficulties and enhance their strengths. Policymakers have the power to fund these initiatives, but providers could advocate with these adolescents and shape a different narrative about them and their lives.

## Figures and Tables

**Table 1:** Sample Characteristics (N = 636).

Variable	Frequency	%
Gender		
Male	290	45%
Female	346	54%
Age		
12–14	118	19%
15–17	428	67%
18–22	89	14%
Government assistance		
Yes	476	76%
No	154	24%
Sexual orientation		
Heterosexual	475	81%
Gay	25	4%
Bisexual	59	10%
Pansexual	7	1%
Transgender	2	0.34%
Other	14	2.40%
Living in the Household		
Two parents	194	31%
Single mother	357	56%
Single father	24	4.0%
Grandfather	24	4.0%
Grandmother	94	15%
Brothers	326	51%
Sisters	324	50%
Legal guardian	54	8.0%
Adoptive Parent	6	0.01%
Other Relative	98	16%

**Table 2:** Bivariate Correlations on Mental Health (N = 548).

Mental Health	1					
School Climate	−0.14 *	1				
Parent Relationships	−0.11 **	0.42 ***	1			
Parent bonding	−0.14 **	0.34 ***	0.56 ***	1		
RACV	0.10	0.23 ***	0.13 *	0.07 **	1	
Community Violence	0.11 **	−0.03	−0.02	0.01	0.31 ***	1
Mean, (SD)	11.86 (12.41)	14.68 (4.16)	3.89 (0.28)	3.85 (1.06)	1.29 (0.58)	1.42 (1.06)
Range	0–61	0–20	0–5	0–5	0–3	0–3

*p* < 0.05 *, *p* < 0.01 **, *p* < 0.001 ***.

**Table 3:** Path Analysis on Mental Health (N = 548).

Observed	B	95% CI	SE	β
Direct Effects				
Structural				
School Climate				
Parent Bonding	0.46 ***	0.10, 0.82	0.18	0.12 *
Parent Relationships	1.39 ***	0.97, 1.81	0.21	0.30 ***
RACV				
School Climate	0.46 **	0.02, 0.07	0.02	0.32 **
Community Violence				
RACV	0.62 ***	0.46, 0.78	0.08	0.33 ***
Mental Health	−0.42 ***	−0.67, −0.17	0.12	
Community Violence	1.22 **	0.26, 2.18	0.49	0.11 *
Parent Bonding	−1.42 *	−2.60, −0.24	0.60	−0.12 *
Parent Relationships	−0.29 ***	−1.67, 1.08	0.70	−0.02
Indirect Effects				
Community Violence				
School Climate	0.03 **	0.01, 0.05	0.01	
Parent Bonding	0.01 *	−0.00, 0.03	0.01	
Parent Relationships	0.04 *	0.01, 0.07	0.02	
Mental Health				
School climate	0.03	−0.00, 0.07	0.02	
RACV	0.76 **	0.13, 1.39	0.32	
RACV				
Parent Bonding	0.02	0.00, 0.04	0.01	
Parent Relationships	0.06 **	0.02, 0.11	0.02	

*p* < 0.05 *, *p* < 0.01 **, *p* < 0.001 ***.
